# A Novel High Performance Liquid Chromatographic Method for Simultaneous Determination of Ceftriaxone and Sulbactam in Sulbactomax

**Published:** 2009-03

**Authors:** Sanjay Mohan Shrivastava, Rajkumar Singh, Abu Tariq, Masoom Raza Siddiqui, Jitendar Yadav, P. S. Negi, Manu Chaudhary

**Affiliations:** *Venus Medicine Research Centre, Hill Top Industrial Estate, EPIP Phase I, Jharmajri (Extn.) Bhatoli Kalan, Baddi, Solan (H. P.), India*

**Keywords:** liquid chromatography, ceftriaxone sodium, sulbactam sodium, tetrabutyl ammonium hydroxide

## Abstract

An isocratic liquid chromatographic method with UV detection at 220 nm is described for simultaneous determination of ceftriaxone sodium and sulbactam sodium in Sulbactomax. Chromatographic separation of two drugs was achieved on a Hypersil ODS C-18 column using a mobile phase consisting of a binary mixture of acetonitrile and tetrabutyl ammonium hydroxide adjusted to pH7.0 with orthophosphoric acid in ratio 70:30. The developed Liquid Chromatographic method offers symmetric peak shape, good resolution and reasonable retention time for both drugs. Linearity, accuracy and precision were found to be acceptable over the concentration range of 125-750 ppm for ceftriaxone sodium and 62.5-375 ppm for sulbactam sodium. The LC method can be used for the quality control of formulated products containing ceftriaxone and sulbactam.

## INTRODUCTION

Ceftriaxone is (6R, 7R)- 3[(acetyl-oxy) methyl]-7-[[2Z)-(2-amino-4-thiazolyl)(methoxyamino)-acetyl]amino]-8-oxo-5-thia-1-azabicyclo[4. 2. 0.]oct-2-ene-2-carboxylic acid (1). Ceftriaxone is a cephalosporin beta-lactam antibiotics used in the treatment of bacterial infections caused by susceptible, usually gram positive organism. The bactericidal activity of ceftriaxone result from the inhibition of the cell wall synthesis and is mediated through ceftriaxone binding to penicillin binding proteins (PBPs). It inhibits the mucopeptide synthesis in the bacterial cell wall. The beta lactam moiety of ceftriaxone binds to caboxypeptidase, endopeptidase, transpeptidase, in the bacterial cytoplasmic membrane. These enzymes are involved in cell wall synthesis and cell division. By binding these, ceftriaxone results in the formation of defective cell walls and cell death. Sulbactam sodium is a competitive, irreversible beta-lactamase inhibitor and has good inhibitory activities against the clinically important plasmid mediated beta-lactamases and most frequently responsible for transferred drug resistance. Both ceftriaxone and sulbactam are listed in the United States Pharmacopoeia (2) and the British Pharmacopoeia (3). To meet the clinical needs, a new combination was developed and consequently for the quality control of the formulation an analytical method was required.

A literature survey revealed that several methods have been used for determination of ceftriaxone sodium which includes Spectrophotometry (4-8), High Performance Thin Layer Chromatography (HPTLC) (9), High Performance Liquid Chromatography (HPLC) (5, 10-15), Capillary Electrophoresis (CE) (16, 17) and differential-pulse adsorptive stripping voltammetry (18). Sulbactam was successfully determined by Spectrophotometry (19), Capillary Isotachophoresis (20), HPLC (21-24) and Gas Chromatography-Mass Spectrometry (GC-MS) (25). Sulbactam along with clavulonic Acid (26, 27), Tazobactam (28), Rifampicin (29) was determined by HPLC. Sulbactam in combination with ampicillin sodium, amoxicillin and piperacillin sodium were determined by UV spectrophotometry and HPLC respectively (30-33).

Sulbactomax* (Ceftriaxone-Sulbactam) is a sterile combination of Sulbactam Sodium and Ceftriaxone Sodium available as dry powder for injection. Sulbactomax* is supplied in strengths equivalent to 187.5 mg, 375 mg, 750 mg, 1.5 g and 3.0 g with Solvent for injection. It is administered as intramuscular and intravenous injection after reconstitution with solvent supplied with the pack. The product Sulbactomax* is manufactured by Venus Remedies Limited, India. Sulbactomax* is a synergistic antimicrobial combination with marked *in vitro* antibacterial activity against a broad spectrum of organisms. Sulbactam not only potentiates the antibacterial activity of ceftriaxone but also exhibits a moderate antibacterial activity. By forming a protein complex with beta-lactamases, Sulbactam irreversibly blocks their destructive hydrolytic activity. Thus, Sulbactam addition extends the spectrum of activity of Ceftriaxone. As Sulbactam also binds with some penicillin binding proteins, sensitive strains are often rendered more susceptible to the Sulbactomax* than Ceftriaxone alone. In bacterial strains that produce either low amounts of beta-lactamase, or none at all, a synergistic effect is observed when sulbactam is associated with ceftriaxone that has a complementary affinity for the target sites. Sulbactomax* is active against all the organisms sensitive/resistant to Ceftriaxone. In addition, it demonstrates synergistic activity (reduction in minimum inhibitory concentrations for the combination versus those of each component) in a variety of organisms. Improved Efficacy as compared to Ceftriaxone alone, Lesser Side Effects, Broader Spectrum Coverage and Improved results of bacterial MIC makes this product unique in the world.

The present communication describes isocratic LC method for simultaneous determination of ceftriaxone sodium and sulbactam sodium, which would be used for the quality control of the formulation developed. The advantage of this method is that by doing one column analysis one can save time. As to the best of our knowledge there is no method present for the simultaneous determination of the combined dosage form. This study achieved satisfactory results in terms of selectivity, linearity, precision and accuracy under simple chromatographic condition.

## EXPERIMENTAL

### Chemicals and reagents

Ceftriaxone sodium and sulbactam sodium reference standards (RS) of United States Pharmacopoeia (USP) were bought from Sigma, United States. Sulbactomax*, a fixed dose combination (FDC) was obtained from manufacturer, Venus Remedies Limited, India. Each vial contains 1 g ceftriaxone and 0.5 g sulbactam. Tetrabutyl ammonium hydroxide (TBAH), acetonitrile and phosphoric acid was of chromatographic grade and was obtained from Merck, Germany. All other chemicals were of analytical reagent grade unless specified.

### Apparatus

Chromatographic separation was performed on Agilent 1200 series liquid chromatographic system equipped with G1311A quaternary pump, Agilent variable UV/vis detector and a G1329A Auto Injector. EZ Chrome Elite software was employed for data collecting and processing.

### Chromatographic conditions

Chromatographic Separation was performed on ODS Hypersil C-18 Stainless Steel column with dimension 250 mm × 4.6 mm, 5μ (Thermo Electron Corporation). The mobile phase consisting of a binary mixture of acetonitrile and TBAH adjusted to pH7.0 with orthophosphoric acid in ratio 70:30, was delivered at rate of 1.5ml per minute. The mobile phase was filtered through 0.45 μm membrane filter (Millipore) and degassed prior to use. Separation was performed at ambient temperature i. e. 25°C and detection was made at 220 nm. The injection volume was 10 μl with a run time of 15 min.

### Preparation of standard solution

Dissolve an accurately weighed quantity of ceftriaxone sodium (RS) 50 mg and sulbactam sodium (RS) 25 mg in mobile phase, and dilute quantitatively and stepwise, if necessary, with mobile phase to obtain a solution having a known concentration of about 500 ppm of ceftriaxone sodium and 250 ppm sulbactam sodium.

### Preparation of sample solution

Transfer about 75 mg of sulbactomax, (ceftriaxone sodium and sulbactam sodium for injection), accurately weighed, to 100 ml volumetric flask. Add mobile phase, swirl to dissolve, dilute with mobile phase to volume and mix.

### Data analysis

For determination of ceftriaxone sodium and sulbactam sodium separately inject equal volumes of the standard preparation and the assay preparation into the chromatograph, record the chromatograms, and measure the responses for the major peaks.

## RESULTS AND DISCUSSION

### Method development and validation

**Figure 1:**
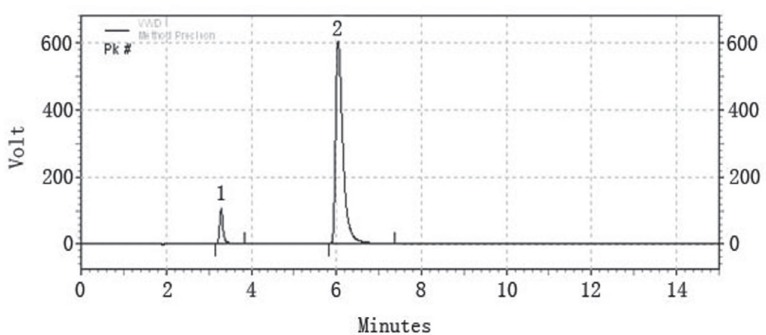
A typical LC chromatogram of a mixture of ceftriaxone sodium, 500 ppm (peak #2) and sulbactam sodium, 250 ppm (peak #1). The chromatographic conditions used were: ODS Hypersil C18 Column, mobile phase of acetonitrile and 0.005N TBAH (30:70), flow rate of 1.5 ml/min, detection wavelength of 220 nm, room temperature 25°C.

Taking in consideration the instability of ceftriaxone sodium and sulbactam sodium in strong alkaline and strong acidic condition, the pH value of the mobile phase should be limited within the range of 3-7. Since mild acidic pH favors the retention and separation of two drugs on C-18 column. After some trials TBAH with pH7.0 was finally selected. Acetonitrile is the most commonly used solvent for LC analysis and often is the first choice for many researchers. Therefore, a binary mixture of acetonitrile and TBAH buffer became the initial mobile phase for the determination of the two drugs. Firstly, various concentrations of TBAH buffer were tried to find the proper one to achieve our purpose. As a result, 0.005N TBAH buffer was found to be ideal for our work. Then, the proportion of acetonitrile and TBAH buffer in mobile phase was determined by varying the proportion of acetonitrile and TBAH buffer from 20:80, 25:75 to 30:70. Finally, the 30:70 ratio of acetonitrile and TBAH buffer was employed for the simultaneous determination of the two drugs, this system produced symmetric peak shape, good resolution and reasonable retention time for both the drugs. The retention times of ceftriaxone sodium and sulbactam sodium for six repetition were 3.426 ± 0.006 and 5.982 ± 0.02 min respectively. The run time is 15 min is taken following the in house standard operating procedure provided for the analysis, which states that the run time should be 2.5 times of the retention time. A typical chromatogram of a sample solution is shown in (Fig. 1).

Since both ceftriaxone sodium and sulbactam sodium in the mobile phase have no significant UV maximum but end absorption, to ensure the sensitivity of the method, the wavelength of 220 nm was employed for the detection.

### Selectivity

**Figure 2:**
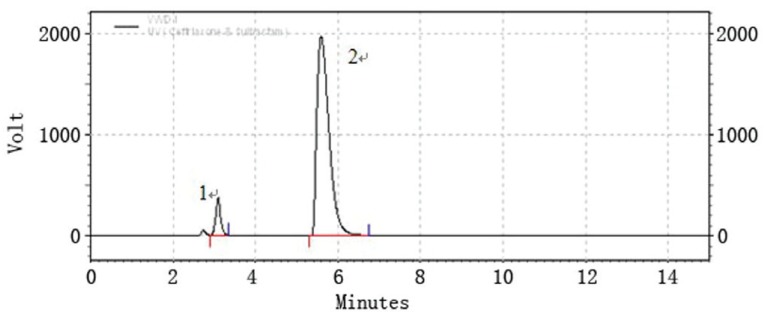
LC chromatogram of ceftriaxone sodium (peak #2) and sulbactam sodium (peak #1); photo-degradation.

**Figure 3:**
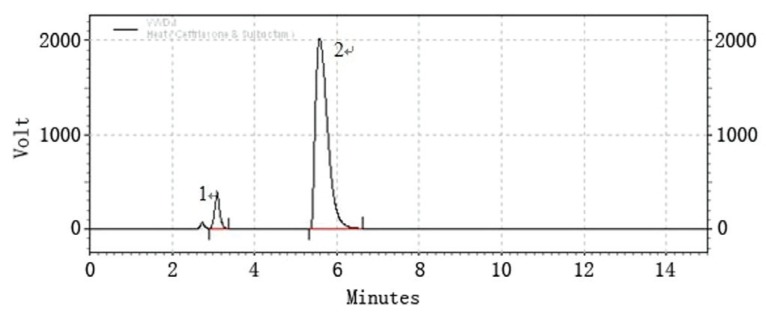
LC chromatogram of ceftriaxone sodium (peak #2) and sulbactam sodium (peak #1); thermal degradation.

**Figure 4:**
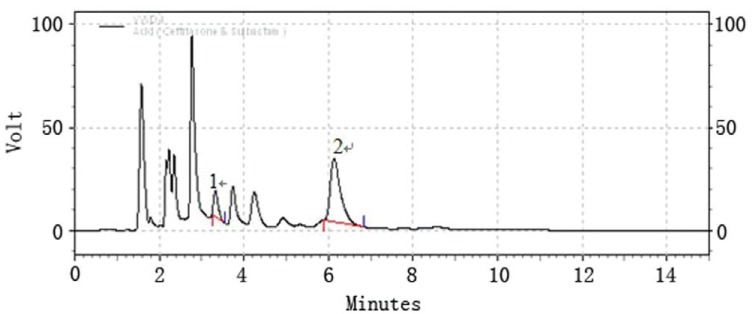
LC chromatogram of ceftriaxone sodium (peak #2) and sulbactam sodium (peak #1); acid degradation.

**Figure 5:**
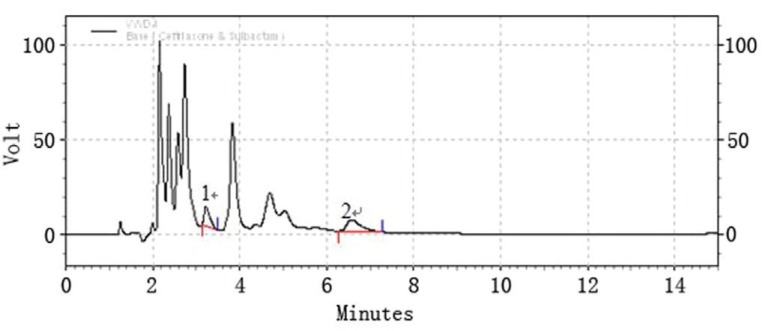
LC chromatogram of ceftriaxone sodium (peak #2) and sulbactam sodium (peak #1); base degradation.

Selectivity is the ability of an analytical method to differentiate and quantify the analyte in the presence of other components in the sample, it can be determined by analyzing forced degraded powder samples. Forced degradation studies were performed to provide an indication of stability property of the proposed method by exposing the formulation product to stress condition of UV light, high temperature (105°C), acid (0.5 M HCl) and base (0.5 M NaOH) in order to test the ability of the proposed method to separate the active component. The samples were degraded to levels where the contents of ceftriaxone and sulbactam in the samples were lowered to that of the original level. Chromatograms for the photo-degradation (UV), thermal degradation, acid and base degradation were individually shown in (Fig. 2-5). Under the given stress conditions, the two drugs are unstable and significant degraded peak appear. The two major peaks can be found in (Fig. 2, 3) but disappear in (Fig. 4, 5). These results suggest that the stability of the two drugs under acidic and basic conditions is poor. For the sake of quantification of peaks obtained after the degradation and to check the formation of impurities the concentration of the product taken is doubled ie 150 mg/100 ml. In case of thermal and photodegradation the product is found to be stable for 4h.

Beside this, placebo formulations were also determined to check the interference from the excipients. A clean chromatogram was obtained, indicating no interference from the excipients. The ability of the method to separate the drugs from their degradation products and non-interference from their excipients indicate the good selectivity of the developed method.

### Specificity

Specificity is the ability of the method to accurately measure the analyte in the presence of all potential sample components. The response of analyte in test mixtures containing analyte and all potential sample components in compound with response of a solution containing only analyte. The analyte peak is evaluated for peak purity from the nearest eluting peak. For this purpose a solution containing 500 ppm of ceftriaxone sodium and sulbactam sodium was injected and peak purity was done. The acceptance criteria for peak purity is that the purity angle should be less than purity threshold. Result of peak purity analysis was found to be satisfactory, purity angle and purity threshold for ceftriaxone and sulbactam is 1.00 and 0.99, respectively.

### System suitability

System performance parameters of the developed HPLC method were determined by analyzing standard working solutions. Chromatographic parameters, such as number of theoretical plates (N), resolution (Rs), capacity factor (k) and selectivity factor (α) were determined. The results are shown in (Table 1), indicating the good performance of the system. System repeatability was determined by five replicate injections of a working standard solution, and the relative standard deviations (RSD) of peak areas of both drugs were calculated to evaluate the repeatability. It was found that RSD for both the drugs was less than 2.0%.

**Table 1:** System performance parameters for ceftriaxone sodium and sulbactam sodium (n=5)

Peak #	Compounds	t_R_ (min)	N	K	R_S_	α

1	Sulbactam	3.426	8966	1.22	--	--
2	Ceftriaxone	5.982	6647	2.88	10.69	2.36

The chromatographic conditions used were: ODS Hypersil C-18 Column, mobile phase of acetonitrile and 0.005 N TBAH (30:70), flow rate of 1.5 ml/min, detection wavelength of 220 nm, room temperature 25°C. t_R_, retention time; N, theoretical plates; K, capacity factor; R_S_, resolution; α, selectivity factor.

### Linearity

**Figure 6:**
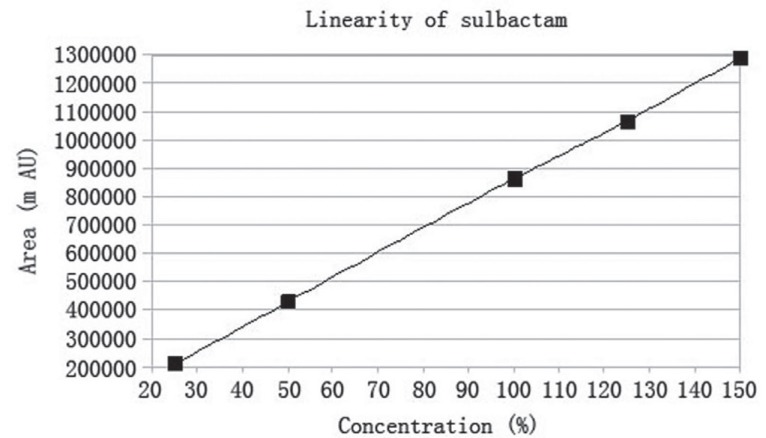
Linearity graph of sulbactam. The chromatographic conditions used were: ODS Hypersil C-18 Column, mobile phase of acetonitrile and 0.005 N TBAH (30:70), flow rate of 1.5 ml/min, detection wavelength of 220 nm, room temperature 25°C.

**Figure 7:**
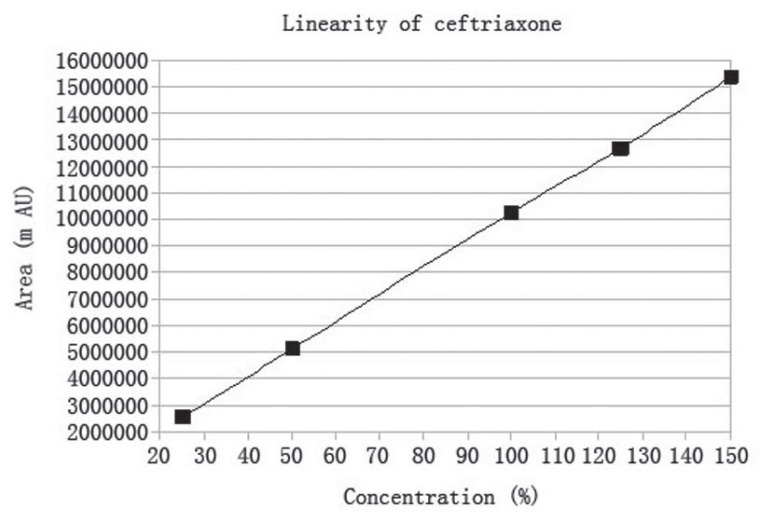
Linearity graph of ceftriaxone. The chromatographic conditions used were: ODS Hypersil C-18 Column, mobile phase of acetonitrile and 0.005 N TBAH (30:70), flow rate of 1.5 ml/min, detection wavelength of 220 nm, room temperature 25°C.

Under the experimental conditions described above, linear calibration curves for both ceftriaxone sodium and sulbactam sodium were obtained with five concentration level each. Peak area (A) and concentration (C) of each drug substance was subjected to regression analysis to calculate the regression equation and the correlation coefficients. The regression equation obtained were A=102337.93 C–6763.23 (r=0.99995, n=5) for ceftriaxone sodium and A=8612.21 C–1152.33 (r=0.99996, n=5) for sulbactam sodium. The individual linearity range was 125-750 ppm for ceftriaxone sodium and 62.5-375 ppm for sulbactam sodium. The results, as illustrated in (Fig. 6, 7) shows that within the tested concentration range there was excellent correlation between the peak area and the concentration of each drugs.

### Limit of detection and limit of quantitation

Limit of detection (LOD) were established at a signal to noise ratio (S/N) of 3.3. Limit of quantification (LOQ) was established at a signal to noise ratio (S/N) of 10 LOD and LOQ were experimentally verified by six injection of ceftriaxone sodium and sulbactam sodium at the LOD and LOQ concentration. The LOD was calculated to be 0.16 ppm for ceftriaxone sodium and 0.49 ppm for sulbactam sodium. And the LOQ was found to be 0.48 ppm for ceftriaxone sodium and 1.49 ppm for sulbactam sodium.

### Accuracy

Accuracy was determined by applying the described method to synthetic mixtures of exipients to which known amount of each drug corresponding to 75,100 and 125% of label of claim had been added. The accuracy was then calculated as the percentage of analyte recovered by the assay. Mean recoveries (mean ± S.D.) for ceftriaxone sodium and sulbactam sodium from the combination formulation are shown in (Table 2) indicating good accuracy of the method for simultaneous determination of the two drugs.

**Table 2:** Accuracy and precision of the HPLC method for simultaneous determination of ceftriaxone sodium and sulbactam sodium

Drugs	Accuracy		Precision		
	Mean recovery ± SD	RSD (%, n=9)	75%	100%	125% label claim

Ceftriaxone	100.88 ± 0.31	0.53	0.34	0.29	0.31
Sulbactam	100.54 ± 0.53	0.31	0.39	0.15	0.44

The chromatographic conditions used were: ODS Hypersil C-18 Column, mobile phase of acetonitrile and 0.005N TBAH (30:70), flow rate of 1.5 ml/min, detection wavelength of 220 nm, room temperature 25°C.

### Precision

System precision is the measure of the method variability that can be expected for a given analyst performing the analysis. Precision of the method was determined with the product. An amount of the product powder equivalent to 75,100 and 125% of label of claim was weighed accurately and assayed in five replicate determinations for each of the three weighing amounts. The results for precision are shown in Table 2, indicating that acceptable precision was achieved for ceftriaxone sodium and sulbactam sodium, as revealed by relative standard deviation data (RSD<2.0% in all of the levels of the two drugs).

### Analytical solution stability

The stability of both the standard and the test was determined by monitoring the peak area responses of the standard solution and a sample solution of ceftriaxone sodium and sulbactam sodium at 0, 6, 12 and 24 hours at room temperature and refrigerator. The results show that there is no significant difference in the area for 24 hour.

### Method application

The validated LC method was applied to the simultaneous determination of Sulbactomax for injection. The three batches of the sample were analyzed and the assay results, expressed as percentage of the label claim are shown in (Table 3). The result indicates that the amount of each drug in the injection corresponds to requirement.

**Table 3:** Assay results for ceftriaxone sodium and sulbactam sodium sterile powder for injection (mean ± S.D.)

Batch No.	Sulbactam sodium	Ceftriaxone sodium

1	100.88 ± 0.31	100.54 ± 0.53
2	99.81 ± 0.23	100.12 ± 0.34
3	99.92 ± 0.61	100.30 ± 0.12

The chromatographic conditions used were: ODS Hypersil C-18 Column, mobile phase of acetonitrile and 0.005N TBAH (30:70), flow rate of 1.5 ml/min, detection wavelength of 220 nm, room temperature 25°C.

## CONCLUSION

The developed LC method with UV-Visible detection offers simplicity, selectivity, precision and accuracy. It produces symmetric peak shape, good resolution and reasonable retention time for ceftriaxone sodium and sulbactam sodium. It can be used for the simultaneous determination of ceftriaxone sodium and sulbactam sodium in the pharmaceutical companies and research laboratories for routine analysis.
